# Simultaneous Quantitation of Isoprenoid Pyrophosphates in Plasma and Cancer Cells Using LC-MS/MS

**DOI:** 10.3390/molecules23123275

**Published:** 2018-12-11

**Authors:** Yashpal S. Chhonker, Staci L. Haney, Veenu Bala, Sarah A. Holstein, Daryl J. Murry

**Affiliations:** 1 Department of Pharmacy Practice, University of Nebraska Medical Center, Omaha, NE 68198, USA; y.chhonker@unmc.edu; 2 Department of Internal Medicine, University of Nebraska Medical Center, Omaha, NE 68198, USA; staci.haney@unmc.edu (S.L.H.); sarah.holstein@unmc.edu (S.A.H.); 3 Department of Pharmaceutical Sciences, Mohanlal Sukhadia University, Udaipur, Rajasthan 313001, India; veenu2bala@gmail.com; 4 Fred and Pamela Buffett Cancer Center, University of Nebraska Medical Center, Omaha, NE 68198, USA

**Keywords:** LC-MS/MS, farnesyl pyrophosphate, geranyl pyrophosphate, geranylgeranyl pyrophosphate

## Abstract

Isoprenoids (IsoP) are an important class of molecules involved in many different cellular processes including cholesterol synthesis. We have developed a sensitive and specific LC-MS/MS method for the quantitation of three key IsoPs in bio-matrices, geranyl pyrophosphate (GPP), farnesyl pyrophosphate (FPP), and geranylgeranyl pyrophosphate (GGPP). LC-MS/MS analysis was performed using a Nexera UPLC System connected to a LCMS-8060 (Shimadzu Scientific Instruments, Columbia, MD) with a dual ion source. The electrospray ionization source was operated in the negative MRM mode. The chromatographic separation and detection of analytes was achieved on a reversed phase ACCQ-TAG Ultra C18 (1.7 µm, 100 mm × 2.1 mm I.D.) column. The mobile phase consisted of (1) a 10 mM ammonium carbonate with 0.1% ammonium hydroxide in water, and (2) a 0.1% ammonium hydroxide in acetonitrile/methanol (75/25). The flow rate was set to 0.25 mL/min in a gradient condition. The limit of quantification was 0.04 ng/mL for all analytes with a correlation coefficient (r2) of 0.998 or better and a total run time of 12 min. The inter- and intra-day accuracy (85–115%) precision (<15%), and recovery (40–90%) values met the acceptance criteria. The validated method was successfully applied to quantitate basal concentrations of GPP, FPP and GGPP in human plasma and in cultured cancer cell lines. Our LC-MS/MS method may be used for IsoP quantification in different bio-fluids and to further investigate the role of these compounds in various physiological processes.

## 1. Introduction

**Figure 1:**
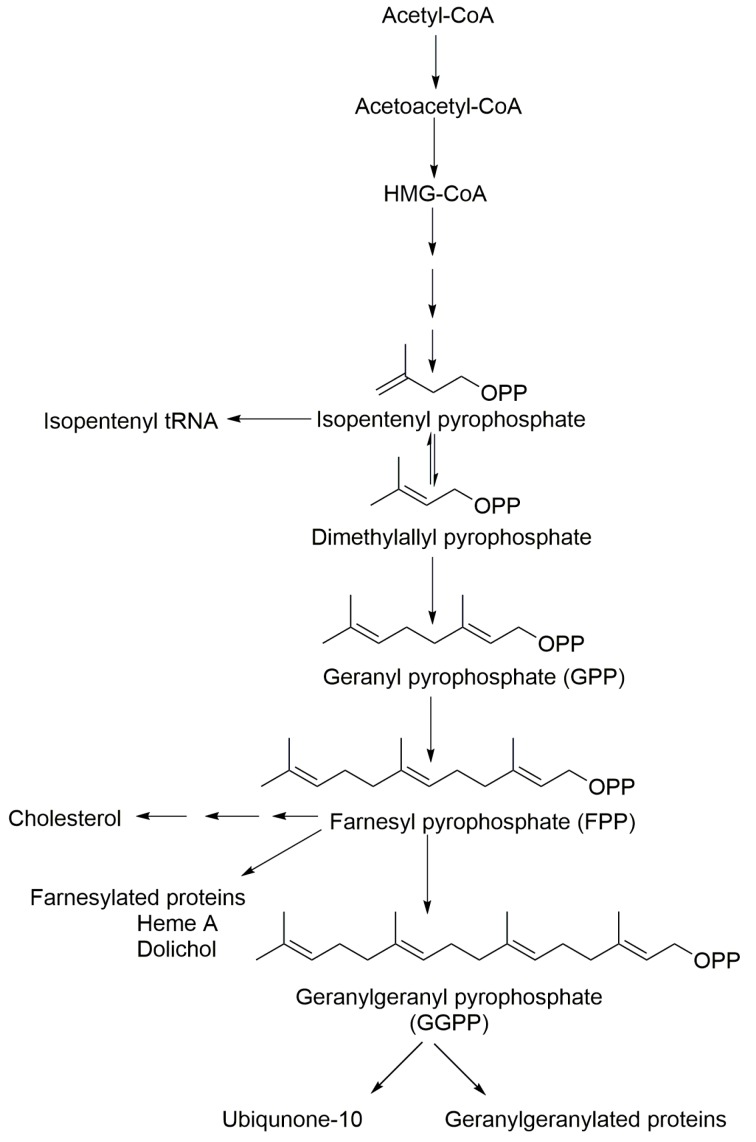
The isoprenoid biosynthetic pathway. Geranyl pyrophosphate (GPP), farnesyl pyrophosphate (FPP), and geranylgeranyl pyrophosphate (GGPP) are key intermediates in the IBP.

The isoprenoid biosynthesis pathway (IBP) plays key roles in cellular metabolism and is responsible for the production of both sterol and non-sterol isoprenoids [1,2]. Because the IBP is an important target in many areas of ongoing research, new inhibitors that target specific enzymes in this pathway are under development. Clinically utilized IBP inhibitors include the statins, which inhibit HMG CoA reductase and are used to treat hyperlipidemia, and the nitrogenous bisphosphonates, which inhibit farnesyl diphosphate synthase (FDPS) and are used to treat a variety of bone diseases including osteoporosis [3], metastatic bone disease [4] and myeloma bone disease [5]. In addition, there is significant interest in the therapeutic potential of inhibiting this pathway in a variety of other diseases, including cancer [2,6,7,8,9], tuberculosis [1], Parkinson’s disease [10,11,12] and hypercholesteremia [13]. Farnesyl pyrophosphate (FPP) and geranylgeranyl pyrophosphate (GGPP) are utilized as the isoprenoid donors for protein farnesylation and geranylgeranylation reactions, respectively. Farnesylation and geranylgeranylation reactions are enzyme-mediated and catalyzed by farnesyl protein transferase (FTase) and geranylgeranyl protein transferases (GGTases) I and II respectively. The membrane localization and function of various proteins, including that of the Ras GTPase superfamily members is facilitated by protein isoprenylation [14]. Therefore, IsoP concentrations in cell culture, plasma and relevant tissues are of great interest in monitoring pathological conditions and modulation following subsequent therapeutic interventions. All isoprenoids share the basic C5 isoprene units, isopentenyl pyrophosphate (IPP) and dimethyl allyl pyrophosphate (DMAPP) and are synthesized in the non-sterol, pre-squalene part of the isoprenoid biosynthesis pathway [15,16]. The head-to-tail condensation of IPP to DMAPP results in the formation of geranyl pyrophosphate (GPP) and the further addition of another IPP produces FPP. GGPP is produced by the condensation of one FPP with one IPP molecule (Figure 1).

Various analytical tools have been developed and validated for the separation and quantification of these IsoPs, including LC–fluorescence detection [17,18,19,20,21,22], and liquid chromatography-mass spectrometry (LC-MS/MS) [23,24,25,26]. LC–fluorescence improves the sensitivity and specificity of IsoP after derivatization but the derivatization process is expensive, time consuming and of limited sensitivity, which makes quantitating IsoP difficult, given that biological concentrations are typically in the pM to nM range. The high sensitivity and selectivity of LC-MS/MS can overcome most of these limitations, which makes it the method of choice for the quantification of IsoP in biological matrices. Hooff et al. reported that high performance liquid chromatography (HPLC) with fluorometric detection allowed the determination of FPP in human brain tissue with a lower limit of quantitation (LLOQ) of 10 ng/mL [18]. Tong et al. reported that HPLC with fluorometric detection following a derivatization process, allowed the determination of FPP and GGPP in tissues [21]. Henneman et al. reported the direct analysis of IsoP in HepG2 cells with an LLOQ of 10 ng/mL [25,26]. Hooff et al. reported a LC-MS/MS method for detecting dansyl-labeled pentapeptide FPP with a LLOQ > 5 ng/mL in human brain tissue [24]. A LC-MS/MS method to detect FPP in human plasma was reported with a LLOQ > 0.02 ng/mL. However, this assay was unable to quantitate GGPP due to interfering peaks [23]. Therefore, a highly sensitive bioanalytical method for determining IsoP in bio-matrices is needed to assess alterations and target the effects in the IBP.

The main challenge in developing a routine quantification method for IsoP analysis in biological samples is that the concentration of these biomolecules in mammalian cells, plasma and tissue are very low and there are several co-eluting interfering endogenous isobaric compounds. Measuring all the intermediates of the IBP pathway in one procedure is a major challenge because the metabolites differ markedly in their structure and physio-chemical properties. Furthermore, chromatographic resolution is required from interfering peaks for their separation and accurate quantification. Here, we report the development of a sensitive (0.04–20 ng/mL) and accurate bioanalytical method using LC–MS/MS, which allows the direct detection and quantification of GPP, FPP and GGPP in bio-matrices without the use of derivatization or fluorescent labels. We also demonstrated the applicability of our method by quantification of individual IsoP in six different human cancer cell lines and human plasma.

## 2. Materials and Methods

### 2.1. Chemicals and Materials

#### 2.1.1. Cell Culture

AsPC-1, BxPC-3, Capan-1, MIA PaCa-2 and PANC-1 cells were obtained from ATCC (Manassas, Virginia, VA, USA) and S2-013 cells have been described previously [27]. The cells were grown in media (RPMI-1640 for AsPC-1, BxPC-3, MIA PaCa-2, S2-013; DMEM for Capan-1, PANC-1) supplemented with 10% heat-inactivated fetal bovine serum, glutamine and penicillin-streptomycin at 37 °C and 5% CO_2_.

#### 2.1.2. Chemicals

Mevalonate (MVA), IPP, GPP, FPP and GGPP were obtained from Sigma-Aldrich (St Louis, MO, USA). Professor David Wiemer at the University of Iowa kindly provided the RAM1147 used as the assay internal standard (IS) [28]. LCMS-grade methanol (MeOH) and acetonitrile (ACN) along with HPLC grade formic acid (FA), ammonium carbonate, and ammonium hydroxide were obtained from Fisher Scientific (Fair Lawn, NJ, USA). Centrifuge tube filters were purchased from Corning Co. (Corning, NY, USA). Oasis^®^ HLB 3cc (30 mg) SPE cartridges (Waters, Milford, MA, USA). Blank plasma (pooled, n = 10) and individual subject plasma was purchased from Equitech Enterprises, Inc., (Kerriville, TX, USA). Ultrapure water was obtained from a Thermo Scientific Barnstead Genpure water purification system (Grand Island, NY, USA). All other reagents were of analytical grade and obtained from standard commercial suppliers.

### 2.2. Liquid Chromatographic and MS/MS Conditions

Sample analysis was conducted using a Shimadzu Nexera ultra high-performance liquid chromatography (UPLC) system (Columbia, MD, USA) equipped with two pumps (LC-30 AD), a column oven (CTO-30AS) and an auto-sampler (SIL-30AC). Peak resolution and separation for all samples were achieved by using an ACCQ-TAG Ultra C18 column (1.7 µm, 100 mm × 2.1 mm I.D., Waters, Milliford, MA, USA) equipped with a C18 guard column. Mass spectrometric detection was performed on an LC-MS/MS 8060 system (Shimadzu Scientific, Inc., Columbia, MD, USA), equipped with a dual ionization source (DUIS) operated in the negative electrospray ionization and multiple reaction monitoring mode in order to achieve unit resolution. The multiple reaction monitoring (MRM) transitions and compound dependent parameters, such as voltage potential Q1, Q3, and collision energy (CE), are shown in Table 1. Optimized parameters were obtained by the product ion scan mode of individual analytes (1 µg/mL) at 0.25 mL/min in methanol/water (50/50, *v*/*v*). Parameters for multiple reaction monitoring (MRM) detection in the negative mode were as follows: Nebulizer gas: 2.0 L/min; heating gas: 10 L/min; drying gas: 10 L/min; interface temperature: 350 °C; desolvation line temperature: 250 °C; heat block temperature: 400 °C. Mass transitions were monitored at 30 ms dwell times and unit mass resolutions and individual parameters are listed in Table 1. Lab Solutions LCMS Ver.5.80 (Shimadzu Scientific, Inc. Columbia, MD, USA) was used for data collection and quantitation.

The mobile phase consisted of a 10 mM ammonium carbonate buffer with 0.1% ammonium hydroxide (mobile phase A, pH 9.7) and 0.1% ammonium hydroxide in acetonitrile/methanol (75/25) (mobile phase B) at a total flow rate of 0.25 mL/min. The chromatographic separation was achieved using a 12 min gradient elution. The initial mobile phase composition was 10% B, increasing in a linear fashion to 65% B over 7 min, then held constant for 2 min, increased to 95% B over 0.5 min, then held constant for 2 min, and finally brought back to the initial condition of 10% B over 0.20 min followed by 1-min re-equilibration. The injection volume of all samples was 10 μL.

### 2.3. Charcoal-Stripped Plasma Preparation

Activated charcoal was used to remove endogenous isoprenoids in the human plasma prior to preparation of standards. Briefly, charcoal suspension (12 mL) was prepared by mixing dextran-coated charcoal (0.66 g) in 100 mL of Dulbecco’s Phosphate buffered saline (DPBS). The sample was centrifuged for 15 min (4000 g at 4 °C) and the supernatant discarded. Plasma (6.0 mL) was then added to the charcoal pellet at 37 ± 1 °C for two hours with continuous stirring, centrifuged at 13,000× *g* for 15 min, and the supernatant collected. The process was repeated twice for maximal removal of endogenous IsoP. This stripped plasma was used to construct plasma calibration curves.

### 2.4. Preparation of Stock, Calibration Standard and Quality Control Sample Preparation

Aliquots from the original stock solutions of GPP, FPP and GGPP were mixed to prepare spiking solution mixtures and stored at −20 °C until used. The stripped plasma and phosphate buffer were used to construct calibration curves for the plasma and culture cell sample analysis, respectively.

The calibration curve (CC) of the analytes ranged from 0.04 to 20 ng/mL. The charcoal-stripped blank plasma or buffer (300 µL) was spiked with mix spiking analyte (10×) solutions to achieve a final standard concentration of 30 µL each, and then vortexed for 30 s. QC samples at four different concentrations (0.04, 1, 5 and 15 ng/mL), the lower limit of quantification (LLOQ), low quality control (LQC), middle quality control (MQC) and high quality control (HQC) were prepared separately in five replicates independently of the calibration standards. A synthetic analog, RAM1147, was used as the internal standard (IS). The IS (10 µL) was added to all CC, QCs and study samples which were then extracted. All the main stocks, intermediate stocks, spiking calibration, and QCs stock solutions were stored at −80 °C.

### 2.5. Plasma Sample Preparation

All samples were prepared by spiking 10 µL of appropriate calibration stock in 300 µL of blank charcoal-stripped plasma and 10 µL of IS solution (1000 ng/mL) were added. Next, samples were diluted with aqueous 2% formic acid in water (900 μL), vortexed for 30 s, and then loaded onto solid phase extraction (SPE) cartridges (Oasis^®^ HLB 3cc, 30 mg; Waters, Milford, MA, USA) pre-conditioned with aqueous 2% formic acid (2 mL). Loaded cartridges were washed with aqueous 2% formic acid (2 mL) and eluted with 2 mL of NH_4_OH:2-Propanol: *n*-Hexane (1:7:12, *v/v*/*v*). For all standards and samples, eluates were evaporated under nitrogen at room temperature and reconstituted in 100 µL of acetonitrile/water (50/50), vortex mixed, and loaded onto the LC-MS/MS for analysis.

### 2.6. Cell Sample Preparation

An aliquot of the cell solution was counted using Trypan blue staining and a Bio-Rad TC20 automated cell counter. Cells were spun down, washed with PBS, and then the cell pellet was stored at −20 °C. To each cell pellet, 0.5 mL of 2-propanol: 100 mM NH_4_HCO_3_, pH 7.4 (1:1 *v/v*), was added, cells were then sonicated, and 300 µL of the resulting cell homogenate was used for further preparation. A 10 µL spiking of the IS was added to the samples, which were then vortexed. Subsequently, 1.0 mL of methanol was added for deproteinization, and then mixture was cooled (-20°C) for 10 min. The samples were then centrifuged for 10 min at 14,000× *g* at 4 °C. After centrifugation, supernatants were transferred to glass tubes and dried under a stream of nitrogen at 40 °C. The residues were then dissolved in 100 µL acetonitrile: water (1:1, *v/v*) and 20 µL of this solution was injected into the LC–MS/ MS system.

### 2.7. Method Validation

Validation procedures for this bioanalytical assay were based on international industry guidelines for bioanalytical method validation by the US Food and Drug Administration (FDA) [29]. Full validation was applied to the human plasma, and partial validation (precision, accuracy, selectivity and recovery) to the buffer.

### 2.8. Selectivity and Specificity

Selectivity and specificity were processed following our extraction procedure. To evaluate selectivity, we analyzed the blank samples spiked with analytes and the IS. Six different lots for blank plasma were evaluated for any interference at the retention times of analytes and the IS to assess the specificity of this method.

### 2.9. Sensitivity

Based on the FDA guidelines, the signal-to-noise ratio (S/N) of the response of the analyte to the calibration standards were used to determine the sensitivity of the method. The S/N ratio was calculated by comparing the base line to the peak response using Labsolution 2.80 software (Shimadzu Scientific, Inc. Columbia, MD, USA). The S/N ratio was required to be greater than three for the limit of detection (LOD) and greater than 10 for the LLOQ.

### 2.10. Accuracy and Precision

Five replicate analyses of the QC samples at four different concentrations (LLOQ, LQC, MQC, and HQC) were prepared on the same day to address intra- and inter-day accuracy and precision data. The precision was calculated in terms of per cent relative standard deviation (% R.S.D.). The criteria for acceptability of the data included accuracy within ± 15% standard deviation (S.D.) from the nominal values and a precision within ± 15% R.S.D. except for the LLOQ, where it could not exceed ± 20% of the accuracy and precision.

### 2.11. Recovery and Matrix Effect

Absolute recoveries of the IsoP and IS from the charcoal-stripped plasma, original plasma, and buffer were calculated by dividing the peak area (after subtracting any endogenous background) from blank samples spiked before extraction to those from neat un-extracted standards for both the LQC and HQC (n = 3).

The matrix effect of human plasma constituents was determined by comparing the responses of the post-extracted plasma standard QC samples (LQC and HQC, n = 3) with the neat standard samples’ response at equivalent analyte concentrations. The peak area ratio was required to be between 85% to 115% to avoid a matrix effect [30,31].

### 2.12. Calibration Curve

Calibration curves were made to determine method linearity by using analyte-spiked charcoalated plasma samples. A blank sample, a zero sample (blank + IS) and 10 non-zero concentrations were prepared to construct each calibration curve. Each back calculated was at a standard concentration with ± 15% deviation from the nominal value except at LLOQ which was set at ± 20% for acceptance.

Two zero samples were injected directly after injecting an HQC sample to check the carry-over. The response of the first zero sample was required to be <20% of the response of a processed LLOQ sample to conclude no carry-over.

### 2.13. Plasma Stability

The stability of the all analytes in the human plasma was studied for LQC, MQC and HQC samples stored in three replicates and recoveries were measured after exposure to different conditions. The auto sampler in plasma and neat samples was studied at 4 °C for 36 h. The bench-top stability was evaluated at 20 °C for 8 h. A storage stability at −80 ± 5 °C for 30 days was also evaluated.

### 2.14. Application of the Method

We also demonstrated applicability of our method by quantification of individual IsoP in six different human cancer cell lines (AsPC1, MiaPaca, BxPC3, Panc1, S2013, and Capan-1). We have also analyzed six plasma samples to measure endogenous concentrations of each individual IsoP.

## 3. Results and Discussion

### 3.1. Chromatographic and Mass Spectrometric Conditions Optimization

**Figure 2:**
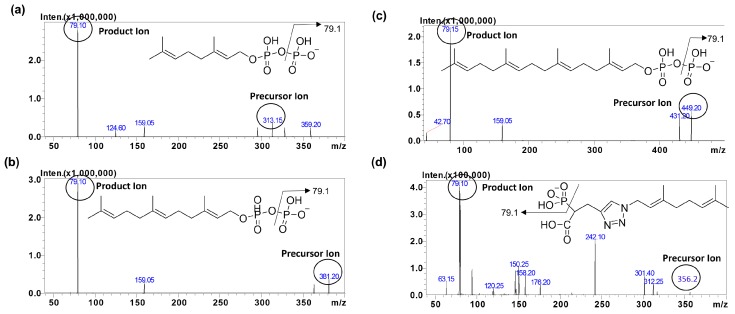
MS/MS product ion spectra of (**a**) GPP, (**b**) FPP, (**c**) GGPP and (**d**) RAM1147 (internal Standard).

The mass spectrometric conditions for detection of IsoP and IS were optimized in the electrospray ionization mode (ESI). To achieve a high selectivity and sensitivity for this method, we optimized our method by comparing electrospray ionization in the negative mode and an atmospheric pressure chemical ionization (APCI) source. ESI in the negative mode was found to offer an improved intensity for the analytes compared to the APCI mode (data not shown). During method optimization, the mass spectra for GPP, FPP, GGPP and IS revealed peaks at *m*/*z* 313.1, 381.2, 449.2 and 356.2, respectively as deprotonated molecular ions, [M − H]^−^. The fragmentation of analytes and IS were auto-optimized via a product ion search of approximately 1000 ng/mL of stock solution for each analyte. The most abundant precursor > product ions for GPP, FPP, GGPP and IS were found to be *m*/*z* 313.1→79.1, 381.2→79.1, 449.2→79.1 and 356.2→79.1, respectively (Figure 2).

Chromatographic conditions were optimized to separate all IsoPs of interest from interference with a desirable peak shape and signal intensity using an ACCQ-TAG Ultra C18, 100 mm × 2.1 mm I.D., 1.7 µm, column (Waters). Different solvent systems such as MeCN and MeOH with various buffers including ammonium carbonate and ammonium bicarbonate with different pH ranges, composition and different flow rates were evaluated. The suitability and robustness of the method were evaluated using different varieties of reverse phase HPLC columns ranging from 50 mm to 150 mm in length (data not shown). The less hydrophobic IsoPs including MVA and IPP eluted early and independently of the mobile phase pH. NH_4_OH (0.1%) was used as an aqueous and organic mobile phase modifier as a basic pH mobile phase resulted in better peak shape and a longer retention of the more hydrophobic IsoPs including GPP, FPP and GGPP. Under our final LC-MS/MS conditions, all IsoPs of interest were resolved from each other as well co-eluting isobaric interferences for target analytes in less than 10 min.

**Figure 3:**
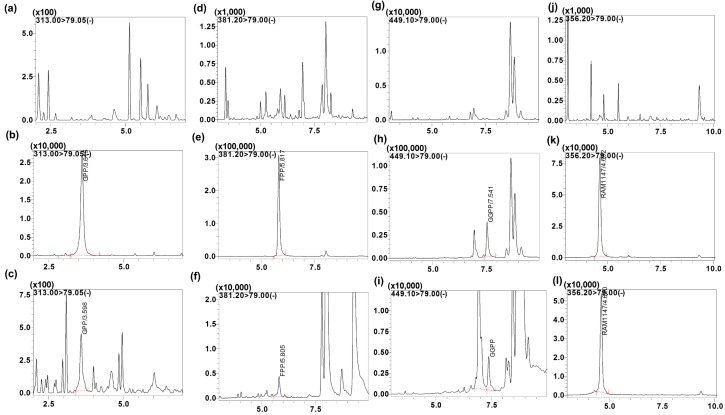
Representative MRM ion-chromatograms of (**a**) blank charcoal-stripped plasma (GPP), (**b**) GPP spiked in charcoal-stripped plasma (rt, 3.6 min,10 ng/mL), (**c**) Blank human plasma sample of GPP, (**d**) blank charcoal-stripped plasma (FPP), (**e**) FPP spiked in charcoal-stripped plasma (rt, 5.8 min,10 ng/mL), (**f**) Blank human plasma sample of FPP, (**g**) blank charcoal-stripped plasma (GGPP), (**h**) GGPP spiked in charcoal-stripped plasma (rt, 7.5 min,10 ng/mL), (**i**) Blank human plasma sample of GGPP, (**j**) blank charcoal-stripped plasma (RAM1147), (**k**) RAM114 spiked in charcoal-stripped plasma (rt, 4.7 min,10 ng/mL), (**l**) Blank human plasma sample spiked with RAM1147.

IsoPs are endogenous compounds and are isobaric with very similar physio-chemical properties. Endogenous compounds not only share the same mass, but also may have the same fragmentation ion (79 is common ion representing the phosphate group), resulting in the same MRM transitions. In addition, interfering peaks could arise from other unknown endogenous matrix components. Therefore, MS/MS specificity is not always adequate for separation of all IsoPs, and chromatographic resolution is required for their separation and accurate quantification. For example, FPP and GGPP had unknown endogenous co-eluting peaks including the same MRM transition but we were able to separate them chromatographically (Figure 3).

Matrix effects became problematic while performing quantification of endogenous analytes including IsoPs. Therefore, it is important to prepare calibration curves in the same or equivalent matrices as of the study samples to minimize the matrix effects. Various approaches have been reported to solve the problem of endogenous background in blank matrices for the construction of calibration curves including background subtraction [32,33], the standard addition method [34], stripping the matrix with charcoal [35,36], and the use of surrogate matrices or stable-isotope labeled standards [37,38,39,40,41,42].

By comparing various calibration approaches, we found that depleting endogenous analytes in human plasma by using activated charcoal was the most accurate and convenient method for this application. Therefore, blank plasma was prepared by stripping plasma from the endogenous IsoP using activated charcoal. The charcoal stripping conditions were optimized to maximize IsoP depletion from plasma. A majority of the IsoP were completely depleted from the charcoal-stripped plasma, however, trace residual peaks for some IsoPs remained even after stripping with charcoal. For these IsoPs, the background peak area was subtracted from the peak area of the calibration curve standards in order to construct the calibration curves with acceptable accuracy and precision. Using analyte/IS peak area ratios, the recoveries of IsoPs in the charcoal-stripped serum were similar to those in unstripped plasma (data not shown), indicating a similar matrix effect for the study samples (unstripped plasma) and calibration curve (stripped plasma).

IsoPs were first isolated from the matrices by solid-phase extractions (SPE) and evaluated using three different SPE cartridges, namely the silica-based C8 and C18 phase cartridge and an Oasis HLB cartridge. The extraction efficiency was improved using an elution combination of NH_4_OH:2-Propanol: *n*-Hexane (1:7:12, *v*/*v*/*v*). We found the stability of the analytes to be influenced by pH, being most stable in basic conditions. Both the desorbing solvent and its volume were investigated to ensure effective elution of the analytes from the sorbent and extraction stability.

The RAM-1147 (structural analogue) was selected as the IS in this method [28]. The IS selected for this method is an isoprenoid triazole bisphosphonate, and therefore serves as a structural analogue to IsoP. The IS had similar chromatographic behavior without prolonging the analysis time, and had a similar ionization response in the ESI mass spectrometry to that of analytes. In addition, the extraction recovery of the IS (>70%) was satisfactory and was stable during the analytical process.

### 3.2. Assay Validation

#### 3.2.1. Specificity and Selectivity

**Figure 4:**
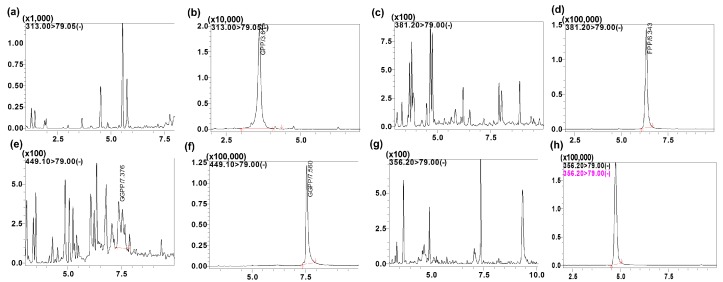
Representative MRM ion-chromatograms of (**a**) blank neat (GPP), (**b**) GPP spiked in neat solution (rt, 3.6 min, 10 ng/mL), (**c**) blank neat (FPP), (**d**) FPP spiked in neat solution (rt, 5.8 min, 10 ng/mL), (**e**) blank neat (GGPP), (**f**) GGPP spiked in neat solution (rt, 7.5 min, 10 ng/mL), (**g**) blank neat (RAM1147), (**h**) RAM1147 spiked in neat solution (rt, 4.7 min, 100 ng/mL).

The specificity of the intended method was established by screening the charcoal-stripped blank plasma from six independent sources. Chromatograms of blank plasma (six batches) contained no co-eluting peaks greater than a peak area of 20% of the analyte area at the LLOQ concentration. There were no co-eluting peaks > 5% of the area of the IS. The retention time for GPP, FPP, GGPP and the IS were 3.6, 5.8, 7.5 and 4.6 min, respectively. The representative UPLC chromatograms with neat blank, standard spiked in the buffer (Figure 4), blank charcoal-stripped plasma, standard spiked in charcoal-stripped plasma at 10 ng/mL concentration, shown in Figure 3 indicating that all endogenous interfering peaks were resolved at the retention times of the analytes. Analytes and IS peak retention time were stable with a relative standard deviation (% R.S.D.) well within the acceptable limit of ±5%.

#### 3.2.2. Accuracy and Precision

Intra- and inter-day accuracy and precision values for the QC samples are determined in order to evaluate the method’s reliability and reproducibility. Accuracy and precision were ≤20% at LLOQ and ≤15% at the other four QC concentrations for all IsoPs in the plasma and buffer. Table 2 shows the inter-day accuracy and precision. These data confirm that the method described has a satisfactory accuracy and precision for the quantitation of all the analytes of interest.

### 3.3. Calibration Curve and Carry-Over

The method was validated for each analyte using three calibration curves prepared on three separate days. The calibration curved ranged from 0.04–20 ng/mL, which encompassed the relevant physiological concentrations [17,23]. For reference, 0.04 ng/mL represents 0.13, 0.10, and 0.09 nM for GPP, FPP and GGPP, respectively. A linear plot of the relation between the concentration and peak area ratio was fitted using a linear regression weighted (1/×^2^). The calibration curve had to have a correlation coefficient (r^2^) of 0.998 or better for all analytes. The analytes showed no significant carry-over effect and there was no significant peak (>20% of the LLOQ) in zero samples injected after the HQC samples.

### 3.4. Recovery and Matrix Effect

The average extraction recovery of all analytes ranged from 40% to 90% in charcoalated plasma and buffer (Table 3). The LQC recoveries of GGPP were slightly low compared to HQC, however not significantly different. In addition, the extraction recovery of RAM1147 (IS) was 70.5 ± 4.9%. Our result confirms charcoal-stripped plasma to mimic human plasma with similar recovery rates using analyte/IS peak area ratios. The matrix effects calculated were in the range of 91.7% to 107.2%. Therefore, ion suppression or enhancement from human plasma was negligible under the current conditions.

### 3.5. Plasma Stability

The results of the stability studies were enumerated in Table 4. In the different stability experiments carried out, including the long-term storage, bench-top and auto sampler, the mean percentage values of the analytes were found to be within ±15% (acceptable limits) of the predicted concentrations for the analytes at their LQC, MQC and HQC levels.

### 3.6. Application of the Method for Samples Analysis

The applicability of our method was demonstrated by the quantitative profiling of endogenous IsoP in six human pancreatic cancer cell lines: AsPC1, MiaPaca, BxPC3, Panc1, S2013 and Capan-1 (Table 5). GPP levels were below the limit of quantitation in Capan-1 and MiaPaca cell lines and could not be quantified. In order to find correlations among the different cancer cell lines, the mean concentrations (given in nmol/10^6^ cells) and standard deviation of isoprenoid concentrations were recalculated in relation to the number of cells in each analyzed sample. A wide variation of the intracellular concentration for each targeted isoprenoid among the different cancer cell lines was observed. The highest concentrations of GPP and FPP were observed in the Panc1 cell lines.

We also analyzed human plasma samples to measure the endogenous concentrations of individual IsoPs. The plasma concentrations of GPP, FPP and GGPP were 0.14 ± 0.02, 0.25 ± 0.09 and 1.66 ± 0.54 ng/mL in human plasma, respectively. The obtained plasma concentrations of FPP (0.25  ± 0.09 ng/mL) in this study were lower than the previously reported value of 1.61 ± 0.40 ng/mL, which may be due to sample variability [24]. Previously, others have not reported human plasma GGPP levels because of the presence of interfering peaks [24]. Our results demonstrate marked variation in the basal concentrations of FPP, GPP and GGPP in human plasma and human pancreatic adenocarcinoma cell lines. The relationship between isoprenoid pool sizes and tumor characteristics is currently not well understood and further research is needed to define this relationship as well as to determine the impact of isoprenoid pool sizes on the response to therapies targeting this pathway.

## 4. Conclusions

A sensitive and specific bioanalytical method to determine the endogenous concentrations of IsoPs in human cancer cell lines and human plasma by LC-MS/MS has been developed and validated. The calibration curve ranged from 0.04 to 20 ng/mL and adequately covered the endogenous concentrations of GPP, FPP and GGPP in pancreatic cancer cell lines and human plasma. This simple, rapid, and reliable method was successfully applied to measure the basal concentrations of GPP, FPP and GGPP in different human pancreatic cancer cell lines and human plasma. The current LC-MS/MS method provides a valuable tool to aid in the understanding of the pathological and physiological role of IsoPs in humans and to determine the effects of IsoP inhibitors.

## Figures and Tables

**Table 1:** Mass spectrometric parameters for individual isoprenoids (IsoPs): precursor to fragment ion transition, voltage potential (Q1), collision energy (CE) and voltage potential (Q3).

Analytes	MRM Transition *m*/*z* (Q1→Q3)	Q1 (V)	CE (V)	Q3 (V)	Retention Time
**GPP**	313.1→79.1	11	25	10	3.6
**FPP**	381.2→79.1	14	35	26	5.8
**GGPP**	449.2→79.1	15	25	22	7.5
**RAM1147**	356.2→79.1	27	55	24	4.6

**Table 2:** Inter-day precision (% RSD) and accuracy for GPP, FPP and GGPP in human plasma and buffer solution.

Bio-Matrix	Analytes	LLOQ (0.04 ng/mL)		LQC (1 ng/mL)		MQC (5 ng/mL)		HQC (20 ng/mL)	
		Accuracy	% RSD	Accuracy	% RSD	Accuracy	% RSD	Accuracy	% RSD
**Charcoalated Plasma**	GPP	97.8	8.2	102.3	3.2	98.3	7.6	102.5	7.8
	FPP	91.9	7.4	92.5	4.7	104.6	8.5	108.4	2.9
	GGPP	100.7	6.2	112.7	8.3	97.2	5.4	91.1	9.1
**Buffer**	GPP	101.2	6.6	105.2	5.8	97.8	10	97.3	1.8
	FPP	110.2	2.5	112.5	7.7	101.3	2.5	95.2	4.9
	GGPP	94.4	7.8	98.4	2.9	105.2	5.4	103.1	7.8

**Table 3:** Mean extraction recoveries (percentages, mean ± SD, n = 3) of GPP, FPP and GGPP from human plasma and cultured cancer cells.

**Analyte**	**Charcoalated Human Plasma**		**Buffer**	
	LQC	HQC	LQC	HQC
**GPP**	55.1 ± 4.2	59.1 ± 5.9	85.1 ± 9.1	81.5 ± 9.9
**FPP**	63.8 ± 5.9	64.2 ± 4.4	87.8 ± 10.5	84.8 ± 8.9
**GGPP**	40.2 ± 4.4	47.3 ± 5.5	74.5 ± 8.7	88.5 ± 5.7

**Table 4:** Mean stability of the GPP, FPP and GGPP at different storage conditions in human plasma.

Analyte	% Stability (Mean ± SD)		
	Auto-Sampler (4 °C, 36 h)	Long-Term (−80 ± 5 °C, 30 days)	Bench-Top (20 °C, 8 h)
**GPP**	96.4 ± 4.6	91.3 ± 7.8	103.4 ± 8.6
**FPP**	93.5 ± 9.6	102.5 ± 9.5	98.5 ± 9.1
**GGPP**	104.4 ± 6.7	93.6 ± 7.3	109.2 ± 5.2

**Table 5:** The concentrations of IsoP endogenous compounds in human pancreatic cancer cell lines.

Cell Lines	GPP	FPP	GGPP
	Concentration (Mean ± SD) (nM/10^6^ cells, n = 3)		
**AsPC1**	0.28 ± 0.08	0.84 ± 0.28	1.96 ± 0.60
**MiaPaca**	BLQ	1.11 ± 0.12	2.31 ± 0.76
**BxPC3**	0.61 ± 0.10	1.49 ± 0.33	2.67 ± 0.75
**Panc1**	0.68 ± 0.11	1.86 ± 0.11	9.96 ± 1.08
**S2013**	0.29 ± 0.08	0.77 ± 0.07	2.00 ± 0.95
**Capan-1**	BLQ	0.50 ± 0.22	0.29 ± 0.06

## Abbreviations

DMAPP	dimethyl allyl pyrophosphate
DMEM	Dulbecco’s modified Eagle’s medium
FPP	farnesyl pyrophosphate
FPPS	farnesyl pyrophosphate synthase
GGPP	geranylgeranyl pyrophosphate
GPP	geranyl pyrophosphate
GGDPS	geranylgeranyl diphosphate synthase
HMG-CoA	3-hydroxy-3-methylglutaryl-CoA
IBP	isoprenoid biosynthetic pathway
IPP	isopentenyl pyrophosphate
IS	internal standard
LOD	limit of detection
LC-MS/MS	high-performance liquid chromatography–tandem mass spectrometry
MVA	mevalonate
MVAP	5-phosphomevalonate
MPD	mevalonate pyrophosphate decarboxylase
MRM	multiple reaction monitoring
